# Obituary

**Published:** 1911-10-21

**Authors:** 


### Obituary.

Brigade-Surgeon Lieutenant-Colonel George Grant, M.B., I.M.S., retired, has died at Bickley, in his seventy-eighth year. He graduated in 1858 from King's College, London, and very shortly after entered the Indian Medical Service, in which he had a long and distinguished career.

The death is announced of Dr. John Nicholas Miller, of Bickley, in his seventy-fifth year. He was a student of University College, London, and graduated at the London University in 1861, taking the M.D. Lond. in 1864. Dr. Miller was for many years surgeon to the Blackheath and Charlton Cottage Hospital.

By the death of Dr. George Thom a prominent surgeon and one of the oldest borough magistrates has been lost to Devonport. His connection with the Royal Albert Hospital, Devonport, dates back to the early seventies, when he was appointed house surgeon to that institution and held office for six years. Later Dr. Thom was elected honorary surgeon, and for many years worked hard for the hospital to which he, in due course, became consulting surgeon. As a magistrate and citizen Dr. Thom was greatly respected in the "Three Towns," and he will be much missed on the Bench on which he served until a few days before his death. Both he and his son, who succeeds him, were students at King's College Hospital.

A sad death in the profession is reported from Australia, Dr. T. L. Gill, a recently qualified graduate of the Melbourne University, having lost his life under curious circumstances. With two other companions he appears to have been overcome by the fumes of carbon monoxide, emanating from a gas stove in a small bath-room. Dr. Gill had finished resident appointments in Melbourne hospitals, and was about to be married and to start in private practice.

The death is announced of Dr. John Highet, of Workington, Cumberland, in his sixty-first year. He was medical officer of health for the Workington Urban District and certifying factory surgeon. Dr. Highet graduated in 1873 at the University of Glasgow, and ten years later took the M.D. degree.

Dr. William Bernard Young, of Auchnasheen, Ross-shire, died on October 3. He graduated at the Edinburgh University in 1874, and was formerly house surgeon at the Ayr Hospital and at the Paisley Infirmary.

